# Sex differences in atrial remodeling and its relationship with myocardial fibrosis in hypertrophic obstructive cardiomyopathy

**DOI:** 10.3389/fcvm.2022.947975

**Published:** 2022-11-30

**Authors:** Xuanye Bi, Yanyan Song, Chengzhi Yang, Yunhu Song, Shihua Zhao, Shubin Qiao, Jinying Zhang

**Affiliations:** ^1^Henan Province Key Laboratory of Cardiac Injury and Repair, Department of Cardiology, The First Affiliated Hospital of Zhengzhou University, Zhengzhou, China; ^2^State Key Laboratory of Cardiovascular Disease, Department of Cardiovascular Disease, Fuwai Hospital, Chinese Academy of Medical Sciences and Peking Union Medical College, Beijing, China; ^3^State Key Laboratory of Cardiovascular Disease, Department of Magnetic Resonance Imaging, Cardiovascular Imaging and Intervention Center, Fuwai Hospital, Chinese Academy of Medical Sciences and Peking Union Medical College, Beijing, China

**Keywords:** hypertrophic cardiomyopathy, myocardial fibrosis, magnetic resonance imaging, left atrial function, sex differences

## Abstract

**Background:**

This study aimed to explore the effect of sex on left atrial (LA) remodeling and its relationship with myocardial fibrosis in patients with hypertrophic obstructive cardiomyopathy (HOCM).

**Methods and results:**

A total of 85 patients with HOCM were enrolled. Myocardial fibrosis was quantified by the collagen volume fraction (CVF) in myocardial samples. The early atrial peak of emptying rate (PER-E) was assessed by LA volume/time (V/t) curves derived from cardiac magnetic resonance (CMR) imaging analysis. The PER-E index was PER-E normalized by left ventricular (LV) filling volume. Patients with HOCM showed a lower PER-E index than healthy controls (*P* = 0.027). Compared with men, the PER-E (*P* < 0.001) and the PER-E indexes (*P* = 0.012) in women were lower. In CVF-stratified subgroups, a sex difference in the PER-E index was eliminated (*P* > 0.05). The CVF was correlated with the PER-E and PER-E indexes in both sexes (all *P*-values were <0.05). In multivariate regression analysis, sex (*P* = 0.007) and CVF (*P* < 0.001) were independently correlated with PER-E (all *P*-values were <0.05).

**Conclusion:**

Patients with HOCM presented LA reverse remodeling. Impaired LA function was more common in female patients with HOCM due to their susceptibility to myocardial fibrosis.

## Introduction

Hypertrophic cardiomyopathy (HCM) is a complex and relatively common genetic cardiac disorder (1). Many patients remain free of clinically significant symptoms and adverse events (2). However, patients with hypertrophic obstructive cardiomyopathy (HOCM) usually present with exertional dyspnea or angina and may experience severe functional limitation and higher HCM-related death risk (3). Left ventricular (LV) diastolic dysfunction (LVDD) is a major reason for these clinical manifestations in HOCM, with myocardial fibrosis as the pathological basis (4, 5). An enlargement of the left atrium (LA), an established marker of LVDD, acts as a compensatory mechanism to modulate LV filling pressure (6). However, recent studies have shown that functional LA changes became evident at the earliest stages of LVDD (7). Despite the increased knowledge of LA function in HCM, its exact relationship with myocardial fibrosis needs further research to be elucidated.

Sex is an important factor contributing to disease heterogeneity. Interestingly, female patients with HCM have been described to show less ventricular remodeling compared with male patients (8, 9), whereas several studies reported more severe diastolic dysfunction, greater likelihood of heart failure progression, and higher mortality in female patients than in male patients with HCM (10, 11). The possible mechanism might be the susceptibility to myocardial fibrosis in female patients with HCM (12). Although LVDD and myocardial fibrosis are closely related to sex, the sex differences in LA remodeling in patients with HOCM remain undetermined.

Therefore, the current study aimed to investigate the effect of sex on LA remodeling and the association between LA remodeling and myocardial fibrosis in patients with HOCM.

## Materials and methods

### Study population

This study was carried out in accordance with the Declaration of Helsinki. Written informed consent was obtained from every patient. The study protocol was approved by the Ethics Committee.

We consecutively recruited 85 symptomatic adult patients with HOCM who underwent surgical myectomy consecutively from 2016 to 2019. All patients underwent a detailed cardiovascular evaluation, including medical history, clinical examination, 12-lead ECG, and cardiac magnetic resonance (CMR) imaging.

Hypertrophic cardiomyopathy was diagnosed by the presence of a non-dilated and hypertrophied LV on CMR imaging in the absence of other diseases that could account for the hypertrophy. Obstructive HCM was defined as a left ventricular outflow tract (LVOT) gradient either ≥40 mmHg at rest and/or ≥50 mmHg during provocation using Doppler echocardiography (13). Patients with severe valvular disease, stages 3–5 of chronic kidney disease (CKD), connective tissue disease, and osteoarthropathy were excluded from the study.

Control myocardium from the LV septal wall was collected at autopsy of nine individuals (6 men/3 women; mean age 45.4 ± 14.3 years) who died from accidents without any cardiac medical history and their hearts showed no signs of macroscopic or microscopic cardiac lesions. CMR images from 35 age- and gender-matched healthy people (21 men/14 women; mean age 45.2 ± 8.5 years) were used as control subjects.

### Cardiovascular MRI

Cardiac magnetic resonance was performed on a 1.5-T magnetic resonance scanner (Magnetom Avanto, Siemens Medical Solutions, Erlangen, Germany). All imaging acquisitions were captured under breath control. CMR images were analyzed using the standard ventricular analysis software (Medis Medical Imaging systems, Leiden, Netherlands). For all patients, septal wall thickness, and posterior and LV end-diastolic dimensions were all determined in the short-axis view (at the midpapillary level). To evaluate functional parameters, cine images were acquired in three long-axis views (LV 2-chamber, 4-chamber, and LV outflow tract) and continuous short-axis planes encompassing the entire LV using a balanced steady-state free precession sequence. Typical parameters include field of view: 320 × 320 mm; matrix: 192 × 224; slice thickness: 8 mm; slice gap: 2 mm; repetition time: 2.8–3.0 ms; echo time: 1.1–1.5 ms; flip angle: 60–70°; bandwidth: 930 Hz; views per segment: 12–20; temporal resolution: 30–55 ms (depending on the heart rate); cardiac phases: 25; SENSE factor: ×2. Epicardial and endocardial borders of the LV myocardium were manually traced during the whole cardiac phase on each cine short-axis image to obtain LV end-diastolic and end-systolic volumes, ejection fractions, and myocardial mass. Myocardial mass was calculated by multiplying the volume of the myocardium calculated at end-diastole by the specific gravity of the myocardium (1.05 g/ml). The end-diastolic volume index, end-systolic volume index, and mass index were indexed to body surface area (BSA). Late gadolinium enhancement (LGE, %) was performed on 67 patients. LGE images were acquired 15 min after intravenous administration of 0.2 mmol/kg gadolinium-DTPA (Magnevist, Schering, Berlin, Germany) using a phase-sensitive inversion recovery-spoiled gradient echo sequence. LGE images were determined automatically by computer counting all hyper-enhanced pixels in the LV myocardium on each of the short-axis images. LGE images were defined as those with image intensities of 6 SDs above the mean of image intensities in a remote myocardial region in the same image.

**FIGURE 1:**
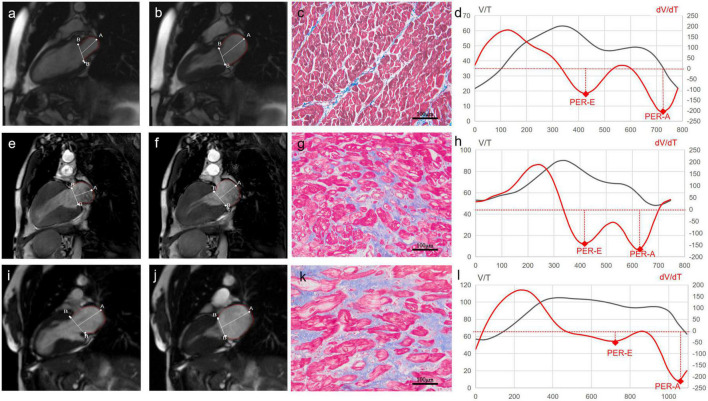
Cardiac magnetic resonance (CMR) images of left atrial (LA) and histological images of myocardium from a control subject (the top panel), a male patient with hypertrophic obstructive cardiomyopathy (HOCM) (the middle panel), and a female patient with HOCM (the bottom panel). The first column is CMR images of the minimum LA volume after atrial contraction in the two-chambered view (Control: **a**, Male: **e**, Female: **i**). The second column is CMR images of the maximal LA volume before the mitral valve opening in the two-chambered view (Control: **b**, Male: **f**, Female: **j**). The third column is the images of myocardial fibrosis (blue) stained with Masson’s trichrome (Control: **c**, Male: **g**, Female: **k**). The fourth column is volume-time curves (black) and their first derivatives (red) of the left atrium (Control: **d**, Male: **h**, Female: **l**). LA V/T curves after the first peak and dV/dt curves under the coordinate axis represent the process of LA emptying. The first negative peak, representing maximal emptying during the conduit phase, is identified as the early atrial peak emptying rate (PER-E). The second peak, during the booster phase, is identified as the late atrial peak emptying rate (PER-A).

Image post-processing was performed using the Tracking Tool software (QStrain version 2.0; Medis Medical Imaging Systems bv). LA endocardial contour was manually traced at the phase of the maximal LA volume before mitral valve opening and at the phase of the minimum LA volume after atrial contraction in the two-chambered and four-chambered views. The atrial and ventricular volume/time (V/t) and dV/dt curves were obtained by plotting the cavity volumes over time (Figures 1d,h,l). From atrial curves, we measured maximum LA volume (LAV max; ml; Figures 1b,f,j) at the end of ventricular systole, and minimum LA volume (LAV min; ml; Figures 1a,e,i) at the end of atrial systole. The left atrial stroke volumes (LASVs; ml) were defined as the difference between the maximal and minimal atrial volumes (i.e., LASV = LAV max-LAV min); the left atrial ejection fraction (LAEF; %) was measured as the ratio in percentage between atrial stroke volumes and maximal atrial volumes (i.e., LAEF = LASV/LAV max × 100%). Peaks of the atrial dV/dt curves were defined as follows: the first negative peak was defined as the early peak empty rate (PER-E; ml/s), and the second peak was defined as the atrial peak empty rate (PER-A; ml/s) representing maximal emptying during the conduit phase and the booster phase, respectively. To be more comparable, LAV max and LAV min were indexed to BSA (m^2^). PER-E and PER-A were also normalized by the LV filling volume (difference between LV end-diastolic and end-systolic volumes), obtaining the PER-E index and the PER-A index. The isovolumetric pulmonary vein transit (IPVT; ml) was defined as the amount of LV filling volume flowing directly from the pulmonary veins into the LV cavity without significant change in LA volume (i.e., IPVT = LV filling volume-LASV). The isovolumetric pulmonary vein transit ratio (IPVTR) was defined as the ratio between IPVT and the atrial emptying volume (i.e., IPVTR = IPVT/LASV), as previously described (14).

### Histomorphological studies

The septal myocardium samples were immediately fixed in 10% buffered formalin and embedded in paraffin. The samples were sectioned and stained with Masson’s trichrome staining for evaluating myocardial fibrosis (Figures 1c,g,k). Four images of every section were acquired with a projection microscope (200×). Subsequent image analysis was performed using the Image-Pro Plus version 6.0 image analysis software (Media Cybernetics Inc., Buckinghamshire, UK) by a cardiovascular pathologist. The extent of myocardial fibrosis was expressed as collagen volume fraction (CVF; %). CVF was calculated as the ratio of collagen-specific staining to the total area of the myocardium in each myocardium sample. The endocardium was excluded from the analysis.

### Statistical analysis

Continuous variables are shown as mean ± SD. Categorical variables are presented as frequencies (percentages). Patients with HOCM were divided into two subgroups according to the upper limit of CVF normality (established as mean + 2 SDs obtained in control subjects and equal to 6%). Of these patients, 51 patients showed high CVF (23 men and 28 women) and 34 patients showed normal CVF (26 men and 8 men). Comparisons of the groups for continuous variables were performed with the unpaired *t*-test or the Mann–Whitney *U* test, whereas the chi-squared test or Fisher’s exact test was used for categorical variables. Pearson’s correlation test or Spearman’s correlation test was used to examine correlations between two continuous variables when indicated. A multivariate analysis was performed with logistic regression analysis using block entry to evaluate if the variables were independent predictors for PER-E, provided to have a *p*-value of <0.1 in a univariate analysis. All *p*-values were two-sided. Statistical analysis was performed using the SPSS software package (version 20; IBM Corp., Armonk, NY, USA).

## Results

A total of 85 patients were enrolled in our study. The baseline clinical characteristics of patients with HOCM are summarized in Table 1.

**TABLE 1:** Baseline clinical characteristics of patients with hypertrophic obstructive cardiomyopathy (HOCM).

	Patients with HOCM			
	All patients	Males	Females	*P*-value
	(*n* = 85)	(*n* = 49)	(*n* = 36)	
Age, years	48.4 ± 13.2	46.2 ± 11.6	51.3 ± 14.7	0.074
Dyspnea, %	79 (92.9)	46 (93.9)	33 (91.7)	1.000
NYHA III/IV, %	30 (35.3)	16 (32.7)	14 (38.9)	0.552
AF, %	13 (15.3)	10 (20.4)	3 (8.3)	0.126
History of hypertension, %	19 (22.4)	8 (16.3)	11 (30.6)	0.12
History of diabetes mellitus, %	8 (9.4)	2 (4.1)	6 (16.7)	0.122
Family history of HCM or SCD, %	6 (7.1)	5 (10.2)	1 (2.8)	0.372
Calcium antagonist, %	24 (28.2)	17 (34.7)	7 (19.4)	0.123
Beta blocker, %	68 (80)	36 (73.5)	32 (88.9)	0.079

AF, atrial fibrillation; HCM, hypertrophic cardiomyopathy; HOCM, hypertrophic obstructive cardiomyopathy; NYHA, new york heart association; SCD, sudden cardiac death.

### Assessment of myocardial fibrosis in patients with hypertrophic obstructive cardiomyopathy

**FIGURE 2:**
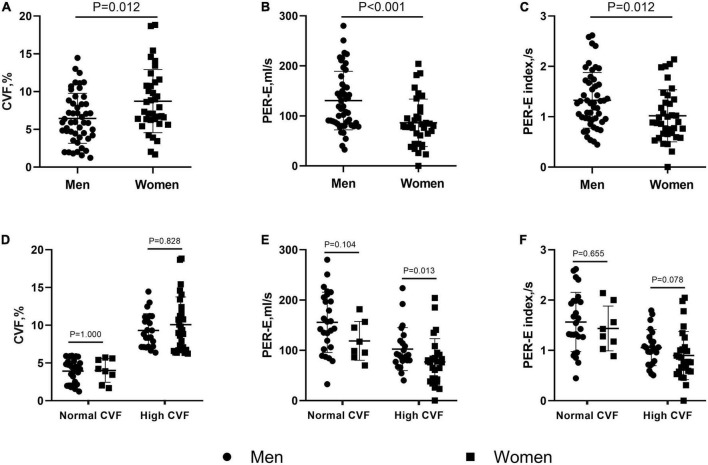
Sex differences in collagen volume fraction (CVF) and left atrial (LA) deformation rates in patients with hypertrophic obstructive cardiomyopathy (HOCM). Female patients showed higher CVF **(A)**, but lower peak of emptying rate (PER-E) **(B)**, and PER-E index **(C)** than males. When stratifying patients by the CVF values, the sex differences in CVF **(D)** and PER-E index **(F)** were eliminated in the subgroups with normal and high CVF, but in PER-E **(E)**, the sex differences remained significant in the subgroup with high CVF.

The CVF values were significantly higher in patients with HOCM than in controls (7.4 ± 3.8% vs. 3.8 ± 1.1%, *P* = 0.002). Women showed more extensive fibrosis than men (8.7 ± 4.2% vs. 6.4 ± 3.3%, *P* = 0.012, Figure 2A). However, no difference in the extent of LGE was found between the two sexes. In subgroups stratified by fibrotic status, there was no difference in CVF between the two sexes (Figure 2D).

### Left atrial structure and function in patients with hypertrophic obstructive cardiomyopathy

Compared with healthy controls, patients with HOCM showed greater septal wall thickness, LV mass index (LVMI), LV end-diastolic diameter (LVEDD), and LV end-diastolic volume index (LVEDVI) (all *P*-values were <0.05). LA diameters, LAV max, and LAV min indices were larger (all *P*-values were <0.005) in patients with HOCM than in the controls. In addition, patients with HOCM had a lower PER-E index and LAEF (1.2 ± 0.6/s vs. 1.6 ± 1.2/s, *P* = 0.027; 41.2 ± 12.2% vs. 59.4 ± 6.6%, *P* < 0.001) than the controls.

As shown in Table 2, LVEDD and septal thickness were lower in women than in men (43.9 ± 4.1 mm vs. 46.3 ± 3.8 mm, *P* = 0.006; 23.9 ± 4.9 mm vs. 26 ± 4.8 mm, *P* = 0.048). However, women showed lower PER-E, PER-E index, and PER-E/PER-A than men (86.3 ± 47.4 ml/s vs. 130.6 ± 58.6 ml/s, *P* < 0.001, Figure 2B; 1 ± 0.5 ml/s vs. 1.3 ± 0.6 ml/s, *P* = 0.012, Figure 2C; 0.64 ± 0.42 vs. 0.97 ± 0.86, *P* = 0.025). The results of the CVF-stratified analyses revealed that female patients with high CVF showed lower LVEDD (43.9 ± 4.4 mm vs. 46.7 ± 3.9 mm, *P* = 0.021), LVMI (89.5 ± 30.6 g/m^2^ vs. 111.1 ± 38.1 g/m^2^, *P* = 0.036), and PER-E (77 ± 46 ml/s vs. 102.3 ± 42.8 ml/s, *P* = 0.013, Figure 2E) than male patients with high CVF. PER-E index (0.9 ± 0.5 ml/s vs. 1.1 ± 0.4 ml/s, *P* = 0.078, Figure 2F), septal thickness (24.1 ± 5 mm vs. 26.4 ± 4.3 mm, *P* = 0.076), and IVPTR (1 ± 0.5 vs. 1.4 ± 1, *P* = 0.082) were lower in female patients than in male patients with high CVF, although the difference was not significant between the two sexes. However, there was no sex-specific difference in CMR parameters in the subgroup with normal CVF.

**TABLE 2:** Cardiac magnetic resonance (CMR) parameters in patients with hypertrophic obstructive cardiomyopathy (HOCM).

				High CVF			Normal CVF		
	Males	Females	*P*-value	Males	Females	*P*-value	Males	Females	*P*-value
	(*n* = 49)	(*n* = 36)		(*n* = 23)	(*n* = 28)		(*n* = 26)	(*n* = 8)	
Left atrium diameter, mm	43.3 ± 8.7	40.6 ± 6.7	0.122	42.3 ± 10.1	41.3 ± 6.3	0.691	44.2 ± 7.4	38.1 ± 8	0.053
LAV max index, ml/m^2^	67.4 ± 26.5	67.1 ± 17.2	0.491	64 ± 25.1	68.5 ± 14.4	0.125	70.3 ± 27.8	62.2 ± 25.5	0.291
LAV min index, ml/m^2^	41.4 ± 23.5	40.5 ± 16.8	0.676	39.3 ± 20.3	41.1 ± 14.3	0.472	43.2 ± 26.3	38.2 ± 24.7	0.372
PER-E, ml/s	**130.6 ± 58.6**	**86.3 ± 47.4**	**<0.001**	**102.3 ± 42.8**	**77 ± 46**	**0.013**	155.7 ± 60	118.7 ± 38.7	0.104
PER-A, ml/s	178.7 ± 85.4	169.9 ± 84.5	0.341	173.4 ± 88.6	175.3 ± 87.8	0.514	183.3 ± 83.8	151 ± 73.7	0.31
PER-E index, /s	**1.3 ± 0.6**	**1 ± 0.5**	**0.012**	1.1 ± 0.4	0.9 ± 0.5	0.078	1.6 ± 0.6	1.4 ± 0.4	0.655
PER-A index, /s	1.8 ± 0.7	2 ± 1.0	0.471	1.8 ± 0.8	2.1 ± 1	0.394	1.8 ± 0.7	1.8 ± 0.8	0.839
PER-E/PER-A	0.97 ± 0.86	0.64 ± 0.42	0.025	0.95 ± 1.1	0.56 ± 0.4	0.135	0.98 ± 0.53	0.91 ± 0.39	0.871
LASV, ml	48.3 ± 18	43.6 ± 13	0.249	45.4 ± 16.3	44.7 ± 13.5	0.985	50.9 ± 19.3	39.9 ± 11.3	0.113
222 LAEF, %	41.1 ± 12.4	41.3 ± 12.2	0.925	41 ± 11.1	41.3 ± 12.1	0.929	41.1 ± 13.6	41.4 ± 13.5	0.957
IPVTR	1.3 ± 0.9	1.1 ± 0.6	0.259	1.4 ± 1	1 ± 0.5	0.082	1.2 ± 0.9	1.2 ± 0.7	0.903
Septal thickness, mm	**26 ± 4.8**	**23.9 ± 4.9**	**0.048**	26.4 ± 4.3	24.1 ± 5	0.076	25.7 ± 5.3	23.1 ± 4.7	0.271
LV end-diastolic diameter, mm	**46.3 ± 3.8**	**43.9 ± 4.1**	**0.006**	**46.7 ± 3.9**	**43.9 ± 4.4**	**0.021**	46 ± 3.8	43.9 ± 3.2	0.165
LVEDVI, ml/m^2^	82.1 ± 19.9	85.3 ± 17.9	0.335	85.9 ± 25.1	83.6 ± 16.8	0.91	78.7 ± 13.6	90.9 ± 21.6	0.208
LVESVI, ml/m^2^	31.5 ± 17.7	30 ± 11.8	0.855	30.8 ± 12.6	29.3 ± 11.3	0.733	32.2 ± 21.4	32.7 ± 14	0.871
LVMI, g/m^2^	99 ± 35.4	91.5 ± 31.4	0.449	**111.1 ± 38.1**	**89.5 ± 30.6**	**0.036**	88.3 ± 29.5	98 ± 35.1	0.626
LVEF, %	64.2 ± 9.8	65.7 ± 8	0.452	64.6 ± 7.7	65.9 ± 8.2	0.586	63.8 ± 11.6	65.1 ± 7.7	0.772
LGE	6.64 ± 4.76	7.08 ± 5.12	0.876	7.18 ± 4.69	8.29 ± 5.36	0.664	6.27 ± 4.86	3.99 ± 2.82	0.227

IPVTR, isovolumetric pulmonary vein transit ratio, defined as the ratio between the PRVT and the atrial emptying volume; LA, left atrial; LAEF, left atrial ejection fraction; LASV, left atrial stroke volume; LAV, left atrial volume; LGE, late gadolinium enhancement; LV, left ventricular; LVEDVI, left ventricle end diastolic volume index; LVEF, left ventricular ejection fraction; LVESVI, left ventricle end systolic volume index; LVMI, left ventricle mass index; PER-A, atrial peak emptying rate; PER-A, index atrial peak emptying rate A normalized by LV filling volume; PER-E, early peak emptying rate; PER-E, index early peak emptying rate normalized by LV filling volume. Bold values mean *P* < 0.05.

### The associations between left atrial cardiac magnetic resonance parameters and myocardial fibrosis in patients with hypertrophic obstructive cardiomyopathy

**FIGURE 3:**
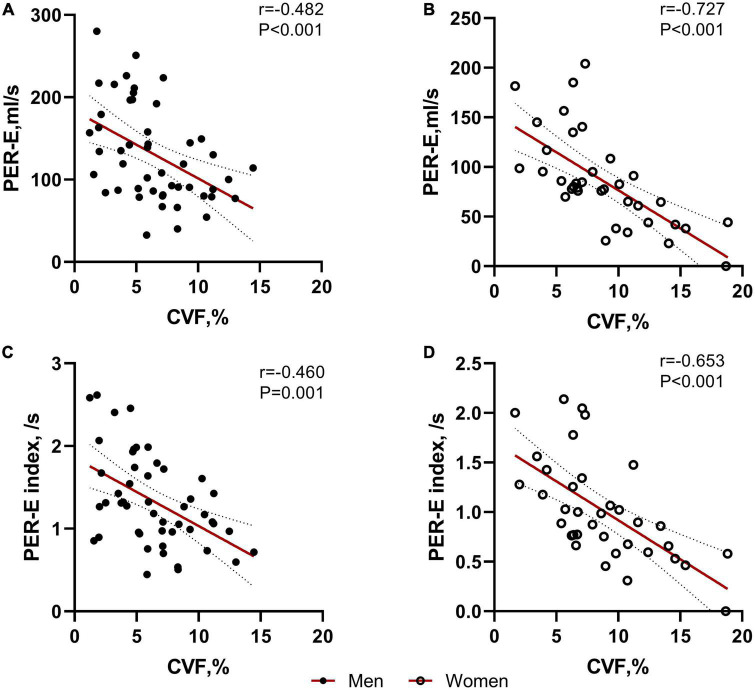
Correlations between collagen volume fraction (CVF) and left atrial (LA) deformation rates in both sexes. CVF was significantly correlated with PER-E and PER-E index in males **(A,C)** and females **(B,D)**.

The CVF value was inversely correlated with PER-E (whole cohort: *r* = −0.604, *P* < 0.001; women: *r* = −0.727, *P* < 0.001, Figure 3B; men: *r* = −0.482, *P* < 0.001, Figure 3A), PER-E index (whole cohort: *r* = −0.568, *P* < 0.001; women: *r* = −0.653, *P* < 0.001, Figure 3D; men: *r* = −0.460, *P* = 0.001, Figure 3C), and PER-E: PER-A ratio (whole cohort: *r* = −0.464, *P* = 0.008; women: *r* = −0.693, *P* < 0.001; men: *r* = −0.269, *P* = 0.061). However, the extent of LGE was not correlated with any LA remodeling parameters.

A univariate regression analysis showed that age (*P* = 0.033), sex (*P* < 0.001), EDVI (*P* = 0.098), ESVI (*P* = 0.071), and CVF (*P* < 0.001) were associated with PER-E. After multivariate adjustment, only sex (*P* = 0.007), EDVI (*P* = 0.012), and CVF (*P* < 0.001) remained significant (Table 3).

**TABLE 3:** Univariate and multivariate regression analyses for peak of emptying rate (PER-E) in patients with hypertrophic obstructive cardiomyopathy (HOCM).

	Univariate		Multivariate	
	β (95%CI)	*P*-value	β (95%CI)	*P*-value
Age	−1.022 (−1.959– −0.084)	0.033		
Sex	44.347 (20.707–67.986)	<0.001	28.341 (8.016–48.666)	0.007
EDVI	0.551 (−0.104–1.207)	0.098	0.658 (0.151–1.165)	0.012
ESVI	0.744 (−0.064–1.552)	0.071		
CVF	−8.914 (−11.582– −6.646)	<0.001	−7.927 (−10.547– −5.308)	<0.001

CVF, collagen volume fraction; HOCM, hypertrophic obstructive cardiomyopathy; LVMI, left ventricular mass index; PER-E, early peak emptying rate.

## Discussion

The major findings can be summarized as follows. (1) Compared with the healthy controls, patients with HOCM showed worse LA remodeling. (2) Female patients were more likely to develop impaired LA deformation rates than male patients. (3) The female sex and myocardial fibrosis were independent predictors for LA deformation rate after adjusting for clinical confounders in patients with HOCM.

Patients with HOCM are characterized by early LVDD, mitral regurgitation, and outflow tract obstruction (15). The abnormal hemodynamics could increase LV filling pressure, leading to LA reverse remodeling. LA function has three phases, serving as a reservoir in systole, a conduit in early diastole, and a booster pump in late diastole. LA reservoir function represents LA relaxation and compliance (16). LA conduit function is reliant on LV diastolic function, including both the suction force dependent on LV relaxation and LV chamber stiffness, whereas LA booster function is based on intrinsic LA contractility and LV end-diastolic compliance and pressure (17, 18). Thereby, there is a close relationship between LA and LV functions.

Cardiovascular magnetic resonance imaging-feature tracking (CMR-FT) is a new quantitative method for wall motion assessment, with a high spatial resolution and large field of view. The details for the change rates of LAV during conduit (i.e., PER-E and PER-E indexes) and booster phases (i.e., PER-A and PER-A indexes) can be provided by the analysis of LA dV/dt curves plotting by CMR-FT. In this study, patients with HOCM showed a lower PER-E index in the conduit phase than controls, but there was no difference in the booster phase. A previous study has also found that patients with non-obstructive HCM were likely to have LA conduit dysfunction, compared to healthy controls (19). The possible explanation is that during early LVDD, increased ventricular stiffness and abnormal relaxation reduce the passive suction effect on LA, which decreases the empty rate in the conduit phase. In contrast, increased atrial stretching results in a more powerful contraction of the LA during the booster phase (6). Therefore, we believed that parameters in the conduit phase are superior as an estimate of the LA function.

In addition, our study showed that the PER-E and PER-E index of female patients is lower than male patients. A previous study has demonstrated that female patients presented more severe atrial stiffness than male patients with heart failure, which was assessed with pulse wave analysis of the radial artery and carotid-femoral pulse wave velocity using commercially available radial artery tonometry. The effect of atrial stiffness can increase LV afterload and impair LV relaxation, which may contribute to a greater susceptibility to heart failure with preserved LV ejection fraction in female patients (20). To explore the relationship between LA remodeling and myocardial fibrosis, we stratified patients with HOCM into two subgroups according to the upper limit of CVF normality. In both subgroups with high and normal CVF, female patients had a similar extent of myocardial fibrosis to male patients. The sex difference in PER-E remained significant in patients with high CVF, but the sex differences in PER-E index were eliminated in both subgroups with normal CVF and high CVF. The multivariate analysis also suggested that sex and myocardial fibrosis were independently correlated with PER-E when adjusting for clinical confounders. This indicated that a higher fibrotic burden in female patients might be one of the factors that led to their worse LA remodeling. Interestingly, at present, LGE imaging is a standard non-invasive approach to evaluate myocardial fibrosis. However, this sex-related difference in myocardial fibrosis could not be found in the LGE analysis, and no correlation was found between the LA function and the extent of LGE. This might be explained by the drawback of detecting diffuse fibrosis in LGE imaging. In patients with aortic stenosis, female patients presented with a larger extent of diffuse myocardial fibrosis but a similar amount of replacement myocardial fibrosis (LGE), which was possible due to the aggressive nature of the response to increasing the LV filling pressure (21). The interaction of sex with myocardial fibrosis was significant in pathological studies (22), but was easily ignored with the use of LGE (23).

The mechanisms underlying sex-specific differences in myocardial fibrosis are unknown, but in our study, despite worse LA function in female patients, they showed smaller LV diameters and lower septal wall thickness. The LV geometry in female patients could underlie a greater prevalence of obstructive hemodynamics (24), which was negatively correlated with LGE (25). Sex hormones and different responses to the renin-angiotensin-aldosterone system may also play a role in the process of fibrosis development (26, 27).

Our study still has several limitations. First, owing to the observational nature of our study, there may be less insight into the causality between LA function and myocardial fibrosis, and it is still unclear how sex modifies the relationship. Second, we only enrolled patients with HOCM, which limited the appliance to other morphological types of HCM. Third, this study is a cross-sectional analysis and may have inherent limitations; the findings must be confirmed by further studies with a longitudinal design.

## Conclusion

Patients with HOCM presented LA reverse remodeling. Impaired LA function was more common in female patients with HOCM due to their susceptibility to myocardial fibrosis.

## Data availability statement

The original contributions presented in this study are included in the article/Supplementary material, further inquiries can be directed to the corresponding authors.

## Ethics statement

The studies involving human participants were reviewed and approved by Ethics Committee of Fuwai Hospital. Written informed consent to participate in this study was provided by the participants’ legal guardian/next of kin.

## Author contributions

XB, YYS, and CY contributed to the conception and design of the study and wrote the first draft of the manuscript. XB organized the database and performed data analysis. All authors contributed to the interpretation of the results and manuscript revision, read, and approved the submitted version.

## References

[1] Maron BJ McKenna WJ Danielson GK Kappenberger LJ Kuhn HJ Seidman CE American College of Cardiology/European society of cardiology clinical expert consensus document on hypertrophic cardiomyopathy. *J Am Coll Cardiol*. (2003) 42:1687–713. 14607462 10.1016/s0735-1097(03)00941-0

[2] Maron BJ. Clinical course and management of hypertrophic cardiomyopathy. *N Engl J Med*. (2018) 379:655–68. 10.1056/NEJMra1710575 30110588

[3] Pelliccia F Pasceri V Limongelli G Autore C Basso C Corrado D Long-term outcome of nonobstructive versus obstructive hypertrophic cardiomyopathy: a systematic review and meta-analysis. *Int J Cardiol*. (2017) 243:379–84. 10.1016/j.ijcard.2017.06.071 28747036

[4] Varnava AM Elliott PM Sharma S McKenna WJ Davies MJ. Hypertrophic cardiomyopathy: the interrelation of disarray, fibrosis, and small vessel disease. *Heart*. (2000) 84:476. 10.1136/heart.84.5.476 11040002 PMC1729476

[5] Avegliano G Politi MT Costabel JP Kuschnir P Trivi M Ronderos R. Differences in the extent of fibrosis in obstructive and nonobstructive hypertrophic cardiomyopathy. *J Cardiovasc Med*. (2019) 20:389–96. 10.2459/JCM.0000000000000800 30994509

[6] Thomas L Marwick TH Popescu BA Donal E Badano LP. Left atrial structure and function, and left ventricular diastolic dysfunction: JACC state-of-the-art review. *J Am Coll Cardiol*. (2019) 73:1961–77. 10.1016/j.jacc.2019.01.059 31000000

[7] Boyd AC Richards DA Marwick T Thomas L. Atrial strain rate is a sensitive measure of alterations in atrial phasic function in healthy ageing. *Heart*. (2011) 97:1513–9. 10.1136/heartjnl-2011-300134 21749989

[8] Leinwand LA. Sex is a potent modifier of the cardiovascular system. *J Clin Invest*. (2003) 112:302–7. 10.1172/JCI200319429 12897194 PMC166308

[9] Schuster A Backhaus SJ Stiermaier T Navarra J Uhlig J Rommel K Left atrial function with MRI enables prediction of cardiovascular events after myocardial infarction: insights from the AIDA STEMI and TATORT NSTEMI Trials. *Radiology*. (2019) 293:292–302. 10.1148/radiol.2019190559 31526253

[10] Borlaug BA Redfield MM Melenovsky V Kane GC Karon BL Jacobsen SJ Longitudinal changes in left ventricular stiffness: a community-based study. *Circ Heart Fail*. (2013) 6:944–52. 10.1161/CIRCHEARTFAILURE.113.000383 23811963 PMC3807873

[11] Chen Y Qiao S Hu F Yuan J Yang W Cui J Left ventricular remodeling and fibrosis: sex differences and relationship with diastolic function in hypertrophic cardiomyopathy. *Eur J Radiol*. (2015) 84:1487–92. 10.1016/j.ejrad.2015.04.026 26001434

[12] Nijenkamp L Bollen I van Velzen HG Regan JA van Slegtenhorst M Niessen H Sex differences at the time of myectomy in hypertrophic cardiomyopathy. *Circ Heart Fail*. (2018) 11:e4133. 10.1161/CIRCHEARTFAILURE.117.004133 29853478

[13] Elliott PM Anastasakis A Borger MA Borggrefe M Cecchi F Charron P 2014 ESC Guidelines on diagnosis and management of hypertrophic cardiomyopathy: the Task Force for the Diagnosis and Management of Hypertrophic Cardiomyopathy of the European Society of Cardiology (ESC). *Eur Heart J*. (2014) 35:2733–79. 10.1093/eurheartj/ehu284 25173338

[14] Aquaro GD Pizzino F Terrizzi A Carerj S Khandheria BK Di Bella G. Diastolic dysfunction evaluated by cardiac magnetic resonance: the value of the combined assessment of atrial and ventricular function. *Eur Radiol*. (2019) 29:1555–64. 10.1007/s00330-018-5571-3 30128617

[15] Maron BJ. Hypertrophic cardiomyopathy: a systematic review. *JAMA*. (2002) 287:1308–20. 10.1001/jama.287.10.1308 11886323

[16] Barbier P Solomon SB Schiller NB Glantz SA. Left atrial relaxation and left ventricular systolic function determine left atrial reservoir function. *Circulation*. (1999) 100:427–36. 10.1161/01.CIR.100.4.427 10421605

[17] Manning WJ Silverman DI Katz SE Douglas PS. Atrial ejection force: a noninvasive assessment of atrial systolic function. *J Am Coll Cardiol*. (1993) 22:221–5. 10.1016/0735-1097(93)90838-r 8509545

[18] Toma Y Matsuda Y Moritani K Ogawa H Matsuzaki M Kusukawa R. Left atrial filling in normal human subjects: relation between left atrial contraction and left atrial early filling. *Cardiovasc Res*. (1987) 21:255–9. 10.1093/cvr/21.4.255 3652092

[19] Yang Y Yin G Jiang Y Song L Zhao S Lu M. Quantification of left atrial function in patients with non-obstructive hypertrophic cardiomyopathy by cardiovascular magnetic resonance feature tracking imaging: a feasibility and reproducibility study. *J Cardiovasc Magn Reson*. (2020) 22:1. 10.1186/s12968-019-0589-5 31898543 PMC6939338

[20] Shim CY Park S Choi D Yang W Cho I Choi E Sex differences in central hemodynamics and their relationship to left ventricular diastolic function. *J Am Coll Cardiol*. (2011) 57:1226–33. 10.1016/j.jacc.2010.09.067 21371640

[21] Tastet L Kwiecinski J Pibarot P Capoulade R Everett RJ Newby DE Sex-related differences in the extent of myocardial fibrosis in patients with aortic valve stenosis. *JACC Cardiovasc Imaging*. (2020) 13:699–711. 10.1016/j.jcmg.2019.06.014 31422128

[22] Lu DY Ventoulis I Liu H Kudchadkar SM Greenland GV Yalcin H Sex-specific cardiac phenotype and clinical outcomes in patients with hypertrophic cardiomyopathy. *Am Heart J*. (2020) 219:58–69. 10.1016/j.ahj.2019.10.004 31726421

[23] Haukilahti MAE Holmström L Vähätalo J Kenttä T Tikkanen J Pakanen L Sudden cardiac death in women. *Circulation*. (2019) 139:1012–21. 10.1161/CIRCULATIONAHA.118.037702 30779638

[24] Nogales-Romo MT Cecconi A Olivera MJ Caballero P Hernández S Jiménez-Borreguero LJ Sex differences in cardiac magnetic resonance features in patients with hypertrophic cardiomyopathy. *Int J Cardiovasc Imaging*. (2020) 36:1751–9. 10.1007/s10554-020-01880-y 32405733

[25] Liu J Zhao S Yu S Wu G Wang D Liu L Patterns of replacement fibrosis in hypertrophic cardiomyopathy. *Radiology.* (2022) 302:298–306. 10.1148/radiol.2021210914 34726536

[26] Greiten LE Holditch SJ Arunachalam SP Miller VM. Should there be sex-specific criteria for the diagnosis and treatment of heart failure? *J Cardiovasc Transl Res*. (2014) 7:139–55. 10.1007/s12265-013-9514-8 24214112 PMC3935102

[27] Patten RD Pourati I Aronovitz MJ Baur J Celestin F Chen X 17beta-estradiol reduces cardiomyocyte apoptosis in vivo and in vitro via activation of phospho-inositide-3 kinase/Akt signaling. *Circ Res*. (2004) 95:692–9. 10.1161/01.RES.0000144126.57786.89 15345655

