# Supplementary material for: Sex differences in atrial remodeling and its relationship with myocardial fibrosis in hypertrophic obstructive cardiomyopathy
Source: Front Cardiovasc Med. 2022 Nov 30;9:947975. doi: 10.3389/fcvm.2022.947975 (PMC9748677; doi:10.3389/fcvm.2022.947975)
Supplement: Supplementary file 1 [file Table_1.DOCX]

| Table S1 CMR parameters in Patients With HOCM and control subjects | | | |
| --- | --- | --- | --- |
|  | HOCM patients | Controls | P value |
|  | (n=85) | (n=15) |  |
| Left atrium diameter,mm | 42.2±8 | 29.2±6.2 | <0.001 |
| LAV max index,ml/m2 | 67.2±22.9 | 36.3±14.4 | <0.001 |
| LAV min index,ml/m2 | 41±20.8 | 14.2±6 | <0.001 |
| PER-E,ml/s | 111.8±58.1 | 105±50.7 | 0.669 |
| PER-A,ml/s | 175±84.6 | 148.2±78.9 | 0.257 |
| PER-E index, /s | 1.2±0.6 | 1.6±1.2 | 0.027 |
| PER-A index, /s | 1.9±0.8 | 2.1±0.8 | 0.549 |
| PER-E/PER-A | 0.8±0.7 | 0.8±0.3 | 0.566 |
| LASV,ml | 46.3±16.2 | 37.8±17.5 | 0.135 |
| LAEF,% | 41.2±12.2 | 59.4±6.6 | <0.001 |
| IPVTR | 1.2±0.8 | 1±0.7 | 0.367 |
| Septal thickness ,mm | 25.1±4.9 | 10.5±2.2 | <0.001 |
| LV end-diastolic diameter,mm | 45.3±4.1 | 48±5.7 | 0.026 |
| LVEDVI ,ml/m2 | 83.4±19.1 | 64.6±12.9 | <0.001 |
| LVESVI ,ml/m2 | 30.9±15.4 | 24.4±5.3 | 0.054 |
| LVMI ,g/m2 | 95.9±33.8 | 38.7±13.4 | <0.001 |
| LVEF ,% | 64.8±9.1 | 62.1±4.9 | 0.093 |
| IPVTR isovolumetric pulmonary vein transit ratio, defined as the ratio between the PRVT and the atrial emptying volume, LA left atrial, LAEF left atrial ejection fraction,LASV left atrial stroke volume,LAV left atrial volume,LV left ventricular, LVEDVI Left ventricle end diastolic volume index,LVEF left ventricular ejection fraction,LVESVI Left ventricle end systolic volume index,LVMI left ventricle mass index;PER-A atrial peak emptying rate ,PER-A index atrial peak emptying rate A normalized by LV filling volume, PER-E early peak emptying rate, PER-E index early peak emptying rate normalized by LV filling volume. | | | |
